# Personalized quantitative models of NAD metabolism in hepatocellular carcinoma identify a subgroup with poor prognosis

**DOI:** 10.3389/fonc.2022.954512

**Published:** 2022-09-30

**Authors:** Adithya Chedere, Madhulika Mishra, Omkar Kulkarni, Shrisruti Sriraman, Nagasuma Chandra

**Affiliations:** ^1^ Department of Biochemistry, Biological Science Division, Indian Institute of Science, Bengaluru, Karnataka, India; ^2^ IISc Mathematics Initiative, Indian Institute of Science, Bengaluru, Karnataka, India

**Keywords:** NAD metabolism, pathway model, NAPRT, NAMPT, liver cancer, precision medicine, patient subtyping

## Abstract

Cancer cells are known to undergo metabolic adaptation to cater to their enhanced energy demand. Nicotinamide adenine dinucleotide (NAD) is an essential metabolite regulating many cellular processes within the cell. The enzymes required for NAD synthesis, starting from the base precursor - tryptophan, are expressed in the liver and the kidney, while all other tissues convert NAD from intermediate precursors. The liver, being an active metabolic organ, is a primary contributor to NAD biosynthesis. Inhibition of key enzymes in the NAD biosynthetic pathways is proposed as a strategy for designing anti-cancer drugs. On the other hand, NAD supplementation has also been reported to be beneficial in cancer in some cases. As metabolic adaptation that occurs in cancer cells can lead to perturbations to the pathways, it is important to understand the exact nature of the perturbation in each individual patient. To investigate this, we use a mathematical modelling approach integrated with transcriptomes of patient samples from the TCGA-LIHC cohort. Quantitative profiling of the NAD biosynthesis pathway helps us understand the NAD biosynthetic status and changes in the controlling steps of the pathway. Our results indicate that NAD biosynthesis is heterogeneous among liver cancer patients, and that Nicotinate phosphoribosyl transferase (NAPRT) levels are indicative of the NAD biosynthetic status. Further, we find that reduced NAPRT levels combined with reduced Nicotinamide phosphoribosyl transferase (NAMPT) levels contribute to poor prognosis. Identification of the precise subgroup who may benefit from NAD supplementation in subgroup with low levels of NAPRT and NAMPT could be explored to improve patient outcome.

## Introduction

**Figure 1:**
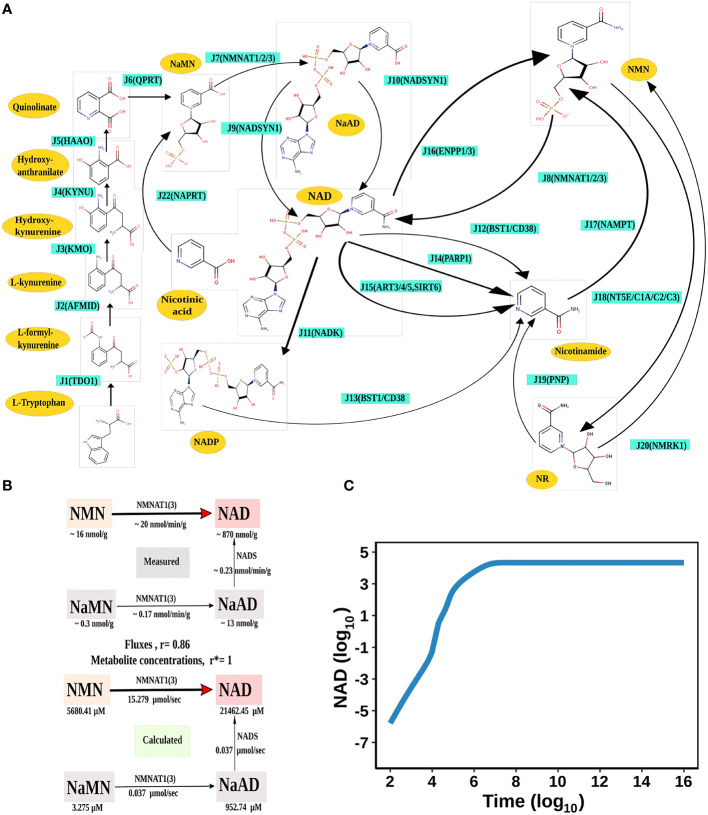
NAD Biosynthesis is perturbed in HCC. **(A)** Diagrammatic representation of the liver NAD_net_. Metabolites are represented as yellow nodes; enzymes are in cyan colour. NAD^+^ can be synthesised by the three different routes - (A) Route I: *de novo* biosynthesis pathway starting with the precursor tryptophan (B) Route II: Preiss−Handler pathway from nicotinic acid and (C) Route III: NAD salvage pathway utilising nicotinamide and nicotinamide riboside for NAD^+^ biosynthesis. Note: NAD represents total NAD in the system (both oxidised and reduced form). **(B)** Validation of the liver NAD_net._ The above panel (Measured) is adopted from the report of Mori *et al.* and represents the metabolic reconstruction of NAD biosynthesis in mouse liver tissue and reflects the main route of NAD generation is *via* NMN. The below panel (Calculated) is the reconstruction of NAD biosynthesis from steady-state concentrations and fluxes obtained from model simulation. r is the Spearman correlation between measured and calculated fluxes. r* is the Spearman correlation between measured and calculated metabolite concentrations. **(C)** Time course simulation of NAD levels for the base model. The blue curve shows the changes in NAD concentration (µM) (log_10_ scale) with respect to time(s) (log_10_ scale).

Nicotinamide adenine dinucleotide (NAD) is an essential cofactor for the cell. It mediates various biological processes such as energy metabolism, DNA repair, signalling, and gene-expression regulation. NAD regulates energy metabolism pathways, including glycolysis, fatty acid oxidation (β-oxidation), the tricarboxylic acid (TCA) cycle, and oxidative phosphorylation (1). NAD exists in both oxidised (NAD^+^) as well as in reduced (NADH) forms; the total concentration of NAD^+^ and NADH is considered as the NAD pool in the cell (2). The NAD^+^/NADH ratio maintains the redox potential of the cell and thereby acts as a metabolic regulator of various NAD-dependent reactions (3). This includes more than 600 metabolic reactions as well as some involved in the signalling. The utilisation of NAD at the global level in the cell makes it an indispensable currency metabolite for the cell (1). Three routes that lead to NAD biosynthesis are well characterised, the first route from tryptophan as a precursor (kynurenine pathway) (**Figure 1A** and **Table 1**: reactions J1-J10), the second from nicotinic acid (Preiss-Handler pathway) (**Figure 1A** and **Table 1**: reaction J22), and the third, a salvage pathway that utilizes several alternative precursors (4–6) (**Figure 1A** and **Table 1**: reactions J8, J17-J20). All the known genes involved in the NAD biosynthesis are expressed in the liver (7–9). In particular, hepatocytes can utilise all precursors from vitamin B3 and from tryptophan to NAD^+^, indicating that the precursors and the synthesis of NAD are high in the liver. The liver also serves as a reservoir of the NAD pool by converting NAD precursors from nutrient sources to nicotinamide (Nam) that can be released into the bloodstream when required (8, 9). Thus, it can be said that the liver regulates the overall physiological requirement of this essential energy currency. NAD does not get degraded in metabolic processes but only interconverts between oxidised NAD^+^ form to the reduced NADH form. On the other hand, processes related to DNA repair, MAPK signalling, Ca^+2^ signalling, and gene expression utilise NAD and degrade it to Nam, which can be later converted back to NAD (7, 10). Hence, any imbalance in the NAD pool will lead to global perturbations in the cell and are known to be associated with various disease conditions such as ageing, inflammation, and cancer.

**Table 1:** NAD_net_ pathway reactions to gene, enzyme, and Factor mappings information.

No.	Reaction ID	Gene	Enzyme name	EC	Reaction	Factor
1	J1	TDO2, IDO2, IDO1	Tryptophan 2,3-dioxygenase	1.13.11.11	L-Tryptophan + O_2_ = N-Formyl-L-kynurenine	F1
2	J2	AFMID	Formylkynurenine formamidase	3.5.1.9	N-Formyl-L-kynurenine + H_2_O = Formate + L-Kynurenine	F2
3	J3	KMO	Kynurenine 3-hydroxylase	1.14.13.9	L-Kynurenine + NADPH + H^+^ + O_2_ = 3-Hydroxy-L-kynurenine + NADP^+^ + H_2_O	F3
4	J4	KYNU	Kynureninase	3.7.1.3	3-Hydroxy-L-kynurenine + H_2_O = 3-Hydroxyanthranilate + L-Alanine	F4
5	J5	HAAO	3-Hydroxyanthranilate 3,4-dioxygenase	1.13.11.6	3-Hydroxyanthranilate + O_2_ = 2-Amino-3-carboxymuconate semialdehyde → Quinolinate	F5
6	J6	QPRT	Quinolinate phosphoribosyltransferase	2.4.2.19	Quinolinate + PRPP = NMN + PPi + CO_2_	F6
7	J7	NMNAT1, NMNAT2, NMNAT3	NMN adenylyltransferase	2.7.7.1	NaMN + ATP = NaAD^+^ + PPi	F7
8	J8	NMNAT1, NMNAT2, NMNAT3	NMN adenylyltransferase	2.7.7.1	NMN + ATP = NAD^+^ + PPi	F8
9	J9	NADSYN1	NAD^+^ synthetase (glutamine-hydrolyzing)	6.3.5.1	NaAD^+^ + ATP + L-Gln + H_2_O = NAD^+^ + AMP + PPi + L-Glu	F9
10	J10	NADSYN1	NAD^+^ synthetase (ammonia-dependent)	6.3.1.5	NaAD^+^ + ATP + NH_3_ = NAD^+^ + AMP + PPi	F10
11	J11	NADK	NAD^+^ kinase	2.7.1.23	NAD^+^ + ATP = NADP^+^ + ADP	F11
12	J12	BST1, CD38	NAD^+^ glycohydrolase	3.2.2.5	NAD^+^ + H2O = Nicotinamide + ADP-ribose	F12
13	J13	BST1, CD38	NAD(P)^+^ nucleosidase	3.2.2.6	NAD(P)^+^+ H2O = Nicotinamide + ADP-ribose(2¢-phosphate)	F13
14	J14	PARP1	Poly (ADP-ribose) polymerase	2.4.2.30	NAD^+^ + (ADP-ribose) _n_ = Nicotinamide + (ADP-ribose) _n+1_	F14
15	J15	ART3, ART4, ART5, SIRT6	Mono ADPribosyltransferase	2.4.2.31	NAD^^+^+^ L-Arg = Nicotinamide + N(2)-(ADP-ribosyl)-L-Arg	F15
16	J16	ENPP1, ENPP3	NAD^+^ pyrophosphatase	3.6.1.22	NAD^+^ + H_2_O = AMP + NMN	F16
17	J17	NAMPT	Nicotinamide phosphoribosyltransferase	2.4.2.12	Nicotinamide + PRPP = NMN + PPi	F17
18	J18	NT5E, NT5C1A, NT5C2	5’-Nucleotidase	3.1.3.5	NMN + H_2_O = Nicotinamide riboside + P	F18
19	J19	PNP	Nicotinamide nucleoside phosphorylase	2.4.2.1	Nicotinamide riboside + P = Nicotinamide + R-1-P	F19
20	J20	NMRK1	Ribosylnicotinamide kinase	2.7.1.22	ATP + Nicotinamide riboside = ADP + NMN	F20
21	J22	NAPRT	Nicotinate phosphoribosyltransferase	2.4.2.11	Nicotinate + PRPP = NaMN + PPi	F22

In cancer, three routes of NAD utilisation are known to be perturbed and are associated with carcinogenesis (7). The first route of NAD utilisation is the NAD mediated central carbon metabolism which is highly altered in cancer (Warburg effect). Reduced values of the NAD^+^/NADH ratio lead to activation of HIF1α through oxidative stress, which in turn activates transcriptional expression of glucose metabolism. Interestingly, not only the central carbon metabolism but other NAD utilising metabolic processes such as fatty acid oxidation, bile acid synthesis, glycerophospholipid metabolism, amino sugar metabolism, etc., are also known to be altered in cancer, especially in hepatocellular carcinoma (HCC) (1, 11). The second utilisation route of the NAD pool is the phosphorylation of NAD^+^ to NADP^+^ by the NAD kinase enzyme (**Figure 1A** and **Table 1**: reaction J11). NADP^+^ also has a variety of cellular functions which are associated with carcinogenesis, such as the reactive oxygen species (ROS) defence, detoxification, and oxidative burst in an immune response. The third and the most important utilisation route of NAD is the NAD degrading ADP-ribose (ADPR) transfer reactions (**Figure 1A** and **Table 1**: reaction J13-J15). NAD acts as a co-substrate for three families of proteins namely Sirtuins, PARPs and cADPR synthases (5). These reactions are critical for CD38 signalling, P53, FOXO, MAPK-dependent growth signalling, mono-ADP-ribosylation reactions, and many more (7). Most of the above-mentioned processes are not only altered in cancer but also have a carcinogenic role in tumour progression. The preferred route of NAD synthesis and utilization is tissue-dependent, and is epigenetically encoded in the cells (5, 10–12). Extracellular NAD pools, partially produced by extracellular NAMPT and NAPRT, aid in inflammation and immune suppression further enhancing the tumour progression (13). The enzymes and metabolites involved in *de novo* pathway are known to be associated with inflammation and immune response (14, 15). Gut bacteria also help in maintaining the NAD pool in the body by producing NAD pathway intermediates, like NaAD, which can be directly converted into NAD (refer to **Figure 1A** and **Table 1**: reactions J9, J10), especially in the liver as well as other organs (16). Therefore, synthesis and NAD utilising reactions, in particular the NAMPT, Sirtuins, and PARPs, have been explored as potential drug targets in the last two decades (17–21). However, many drugs targeting NAD and associated pathways have failed in clinical trials for various reasons such as drug toxicity, patient heterogeneity, and alternate routes of signalling (7, 22).

On the other hand, reports from various epidemiologic studies suggest an association between low NAD precursor diets with an increased rate of cancer incidence (23). NAD levels decrease with ageing, thus forming an association with diseases related to ageing, such as neurodegenerative diseases, cardiovascular diseases, bone dysfunctions, and cancer (24–26). Studies using mice models of cancer and ageing also exhibit low NAD levels and therefore are more prone to oxidative stress. Current knowledge about this suggests that a low NAD level can lead to oxidative stress-induced DNA damage and thereby promote mutagenesis and tumour initiation (10, 27). Thus, a high level of NAD can have a preventive role in tumorigenesis. Another recent study Tummala et al. reported that an increased expression of unconventional prefoldin RPB5 interactor (URI) leads to AhR- and/or ER-mediated reduction of the NAD pool and thereby promotes HCC tumorigenesis due to increased DNA damage (28). This seemed to suggest that NAD supplementation can have a protective role against HCC development and progression in cirrhotic patients (29, 30).

However, while on the one hand, an increase in NAD related activities are linked to metabolic and signalling alterations in cancers, leading to the hypothesis that the pathway is an attractive drug target for tumour killing. On the other hand, NAD protects cells from DNA damage and is found to be downregulated in many cancers including HCC, hence suggesting that supplementation of NAD can stop carcinogenesis. These seemingly opposite findings of NAD imbalance in HCC have led to a controversy about the role of NAD in tumorigenesis. Therefore, it is important to first address the role of NAD in tumorigenesis in individual HCC patients and understand whether it acts as a tumour suppressor or a tumour promoter.

The main objective of this study is to decipher the role of NAD in HCC and to understand whether NAD biosynthesis inhibition or alternately NAD supplementation will be beneficial in treating HCC. To address this objective, it is important to understand the NAD pathway profile in HCC and whether there is any heterogeneity among HCC patients. Pathway modelling offers a useful method to profile the individual enzymes in the pathway and decide whether it is altered in a given individual as compared to the normal liver (NL) and, if so, in which direction. Knowledge of the NAD pathway profile in individual HCC patients will enable precision targeting and ultimately aid the clinician in decision-making for the management of HCC. To answer these questions, in this work, a quantitative kinetic model of the NAD biosynthetic pathway (NAD_net_) is constructed and simulated for each individual patient by integrating patient-specific transcriptomics data available through TCGA-LIHC.

## Materials and methods

### Model building of NAD_net_

#### The base structure of the network

The liver NAD_net_, a liver-specific NAD biosynthesis network, was reconstructed in the laboratory using the previously published NAD model on glioma from our laboratory (31). The model comprehensively captures known reactions in the NAD biosynthesis, covering the *de novo* pyridine ring formation *via* the kynurenine pathway, the utilisation of dietary precursor nicotinate through the Preiss−Handler pathway, and the utilisation of nicotinamide and nicotinamide riboside through the NAD salvage pathway for NAD^+^ synthesis in cancer. A base model was first constructed by considering reactions that can occur in any human tissue, which was subsequently curated to check for the feasibility of each reaction in the human liver. It was observed that out of 24 enzymatic reactions from the base model, only 21 enzymatic reactions were feasible in the liver and were therefore retained. Other than the enzymatic reactions, four non-enzymatic sink reactions were added to the model for the model stability. A list of enzymatic reactions in the model is provided in **Table 1**. Kinetic parameters for each of the enzymes in the model were manually curated from the primary literature. K_cat_, K_m_, and K_i_ for inhibitory interactions were also obtained from the same primary sources, and wherever possible, the parameters for the liver tissue were used. A full list of parameters is provided in **Supplementary Table 1**. A list of fixed metabolites for simulation purposes is provided in **Supplementary Table 2**.

#### Estimation of F_Kcat_ and F_conc_

One of the most challenging tasks in kinetic modelling is to deal with various types of inconsistencies in units of reported parameters. In order to get all parameters in a comparable framework, a factor (F_Kcat_) was calculated so as to represent K_cat_ in 1/sec units for all enzymes. The detailed calculation of (F_Kcat_) factor for each type of specific activity is provided in **Supplementary Table 3**. For each enzyme in the model, enzyme concentration was estimated from the PaxDB database (32). Then, using these estimated K_cat_ values and enzyme concentrations, V_max_ for each reaction was calculated. The estimated V_max_ values are listed in **Supplementary Table 3**.

### Transcriptome data: TCGA dataset

The Cancer Genome Atlas (TCGA) Liver Hepatocellular Carcinoma (LIHC) RNASeq HT-Seq gene expression (counts data) and phenotype data were collected through UCSC Xena (http://xena.ucsc.edu) (TCGA-LIHC cohort) (33, 34). The dataset contains 374 HCC tissue biopsy samples, out of which three are samples from recurrent HCC samples, and the rest are from primary HCC. The dataset included 50 normal liver (NL) tissue biopsy samples as well. For this analysis, we have considered only primary HCC and Normal liver samples. RNASeq counts data was normalised using edgeR package (V 3.34.1) (35). The mean of normalised gene count was calculated for all normal samples and was used as a control to calculate the fold change of each gene for each tumour sample. All ensembl ids were mapped to gene symbols using org.Hs.eg.db package in R Language (V 3.15.0) (36).

### Integration of gene expression data into the model

Fold change values of gene expression of each enzyme were integrated into the model as described previously (37). The correlation between RNA and protein is ~ 0.5, indicating that the transcript levels of genes and the corresponding proteins follow the same trend in their concentrations, justifying the use of RNA levels an indicator for the protein levels (38). For reactions catalysed by multiple genes, the cumulative sum of fold change values in the expression of all associated genes was used. The F-factor for each reaction across all patients was calculated using the mean expression profile of Normal Liver (39). The F-factors differ between the NL model and any patient model. For the NL model, F-factors are all equal to one. For patient models, F-factors are substituted as the cumulative sum of FC values of genes involved in the corresponding reaction. The changes in F-factor values influence the reaction rates. For a given reaction, if the F-factor value is greater than one, the reaction rate is increased by the F-factor value times as compared to the NL model; similarly, if the F-factor value is less than one, the reaction rate is decreased by the F-factor value times as compared to the NL model.

### Mutation analysis

Pre-processed mutation data for each cancer type was obtained from the cBioPortal resource (40). From this, mutation frequencies of genes from the NAD_net_ were retrieved.

### ODE simulation

Ordinary differential equations (ODE) of the reconstructed models were solved to obtain steady-state values using the getSS function, with the modified option of resolution to 1E-03 and the maximum duration for forward integration to 1E+20, in the CoRC (V 0.11.0) package (41) in R (V 4.1.3) (42). Steady-state metabolite concentrations and reaction flux values were analysed.

### Sensitivity analysis

Sensitivity analysis provides a measure of how much a selected model variable (the effect) changes when a selected parameter (the cause) is changed. Sensitivity was calculated for the perturbation effect of individual parameters on the steady-state concentration of NAD. Therefore, it can identify parameters having an effect on NAD concentration. For models which gave results for steady-state analysis, parameter sensitivity analysis was performed. In the current model, there are 114 parameters, so each parameter for a given simulation was only varied by 1 % from the original value, thus resulting in a total of 229 simulations for each model, i.e., one unaltered parameter simulation and 2*114 single parameters altered either by +1 % or -1 % of the original parameter value. All the altered parameter simulations were scaled by taking the percentage change compared to the unaltered parameter simulation. Results were summarised separately for concentrations of metabolites and flux of reactions in the form of a 2D plot with a colour scale representing the percentage change in the simulation value, using the corrplot (V 0.92) package in R (43). The red colour represents an increase in the concentration of the metabolite as compared to the unaltered parameter simulation, whereas the blue colour represents a decrease in concentration of the metabolite. The extent of colour filled in the squares represents the extent of percentage change in the metabolite due to the change in the parameter value.

For a summary of all patient model changes, the percentage change in the altered parameter simulation is calculated and represented as a pie chart of the percentage of models with alterations among all the steady-state models.

### Correlation analysis

The correlation analysis was performed between NAD genes and NAD metabolites using Pearson correlation (log_2_FC values - for numeric variables) in cor.test function from the stats package in R. Corrplot function from the corrplot (V 0.92) package was used to represent the correlogram (43).

### Clustering and heatmap

Hierarchical clustering of gene expression data and metabolite steady-state values with the patient profiles was carried out using the Heatmap function in ComplexHeatmap (V 2.8.0) package in R (44). Gene expression data and calculated metabolite fold changes were categorized into three groups; Up (log_2_FC ≥ +1), No_change (-1 < log_2_FC < +1) and Down (log_2_FC ≤ -1); and substituted with an integer value before Hierarchical clustering; Up (+1), No_change (0) and Down (-1).

### Survival analysis

Survival analysis and univariate Cox regression analysis were performed using the survival (V 3.3-1) package in R (45, 46). Genes and metabolites for each patient were classified as upregulated ( log_2_FC ≥ +1) or downregulated (log_2_FC ≤ -1). Hazards ratio values (HR) were obtained using the coxph function from the survival package. HR in survival analysis is the hazard ratio which essentially is the ratio of the hazard rates corresponding to the conditions described by two levels of gene expression. If the gene has a value HR > +1, the given gene is a poor prognostic marker (over-expression of the gene is associated with high mortality of the patients) and vice-versa. The survdiff function from the survival package was used to identify the significant genes/metabolites (p-value < 0.05) associated with patient survival. Survival plots were generated using the ggsurvplot function from the survminer (V 0.4.9) package (47). The survival analysis was performed among the distinct groups.

## Results

### NAD biosynthesis is perturbed in HCC

#### NAD biosynthesis network in the human liver

The first objective was to reconstruct a NAD biosynthesis network that captures the physiological processes in the human liver tissue. The liver NAD_net_ model consisting of 26 reactions, 29 genes, 31 metabolites, and 138 parameters (**Figure 1A**) was reconstructed using the model published in Padiadpu et al. (31). The liver NAD_net_ model retains all three routes of NAD^+^ biosynthesis - (a) Route I - production of NAD^+^ from tryptophan through the kynurenine pathway, which is known to be active in the liver (b) Route II - utilisation of Nicotinic acid (Na) as a substrate for NAD^+^ generation through the Preiss–Handler pathway and (c) Route III - the salvage pathway of synthesising NAD from extracellular precursors provided by the diet (for, e.g., Nicotinamide (Nam) and nucleosides (Nicotinamide riboside (NR) and Nicotinic acid riboside (NAR)) (4–6, 48). Nam, Na, NR, and NAR are collectively referred to as Vitamin B3. Detailed information about enzymatic reactions is given in **Table 1** and **Supplementary Table 1**. A steady-state analysis of the NAD_net_ was performed using CoRC (V 0.11.0). Steady-state values of metabolites and fluxes of the corresponding reactions are given in **Table 2**. Kinetic stability analysis of the model revealed that it was asymptotically stable.

**Table 2:** Steady-state concentration of metabolite and fluxes of reaction.

A) Steady-state metabolite concentrations		B) Steady-state fluxes of reactions	
Metabolite	Concentration (μM)	Reaction ID	Flux (μM/s)
L-Tryptophan	1.60E + 01	J1	1.90E-03
L-Formyl-kynurenine	6.12E + 01	J2	1.90E - 03
O_2_	1.00E + 03	J3	1.90E - 03
Hydroxy-L-kynurenine	1.34E - 01	J4	1.90E - 03
NADPH	4.00E + 02	J5	1.90E - 03
Hydroxyanthranilate	6.06E - 07	J6	1.90E - 03
Quinolinate	6.70E - 01	J7	3.74E - 02
NaMN	3.28E + 00	J8	1.53E + 01
PRPP	1.00E + 03	J9	3.63E - 02
PPi	1.54E + 04	J10	1.11E - 03
NaAD	9.53E + 02	J11	1.59E - 02
NAD	2.15E + 04	J12	1.70E - 08
NMN	5.68E + 03	J13	1.98E - 11
ATP	1.00E + 03	J14	9.88E + 00
NH_3_	1.00E+02	J15	5.03E + 00
Glutamine	6.00E + 02	J16	3.72E - 01
NADP	1.59E + 01	J17	1.49E + 01
Nam	4.98E + 04	J18	4.81E - 12
ADPribose	1.70E - 05	J19	2.75E - 24
ADPriboseP	1.98E - 08	J20	4.81E - 12
L-Kynurenine	9.21E - 02	J22	3.55E - 02
Arginine_protein	1.00E + 03	re23	2.15E - 02
NR	4.12E - 10	re25	1.59E - 02
P	1.00E + 03	re26	1.54E + 01
R1P	5.00E + 01	re27	1.98E - 11
Na	1.00E + 01	re28	1.70E - 08

#### Validation of NAD_net_

The liver NAD_net_ was first inspected for validity by (a) Steady-state metabolite concentrations from the simulations were compared with the experimentally determined values reported in the literature (8); (b) Steady-state fluxes of enzymes of the enzymatic reactions were compared with the experimentally determined values. The available experimental data about metabolite concentrations were from diverse sources, including liver tissue, blood, and cerebral fluid. Moreover, some were from humans, and some from mice and other model organisms. To add to this difficulty, some were reported as nmol/gm while some others were given as μmol/gm of the liver tissue, making direct comparisons difficult. To overcome this problem, a rank-based correlation, using the Spearman correlation metric, was calculated for both experimental and simulated data. Experimentally constructed NAD biosynthesis rate for mouse liver reported by Mori et al. (8) was compared with the model-predicted metabolite level and flux rate for human hepatocytes (**Figure 1B**). A relative ranking of the metabolites (NAD, NaAD, NaMN and, NMN) and separately of the fluxes obtained from the experimental and from the simulation profile were used to compare the correlation between experimental and computational predictions. For the metabolites, the correlation was found to be +1, and for enzymatic reactions, it was found to be +0.86, suggesting that the model is consistent with experimental data. Utilisation of NMN through NMNAT1 (3) is seen to be the main route of NAD biosynthesis in the liver (**Figure 1B**). Further time-course analysis was performed on the NAD_net_, and the NAD time course was plotted (**Figure 1C**) and compared against experimentally determined time course after labelled NAD supplementation in a mouse model (9). The time course profiles of NAD were in excellent agreement with that reported in the Liu et al. model.

### HCC patients exhibit heterogeneity in their NAD profiles

#### Construction of personalised NAD_net_ models

**Figure 2:**
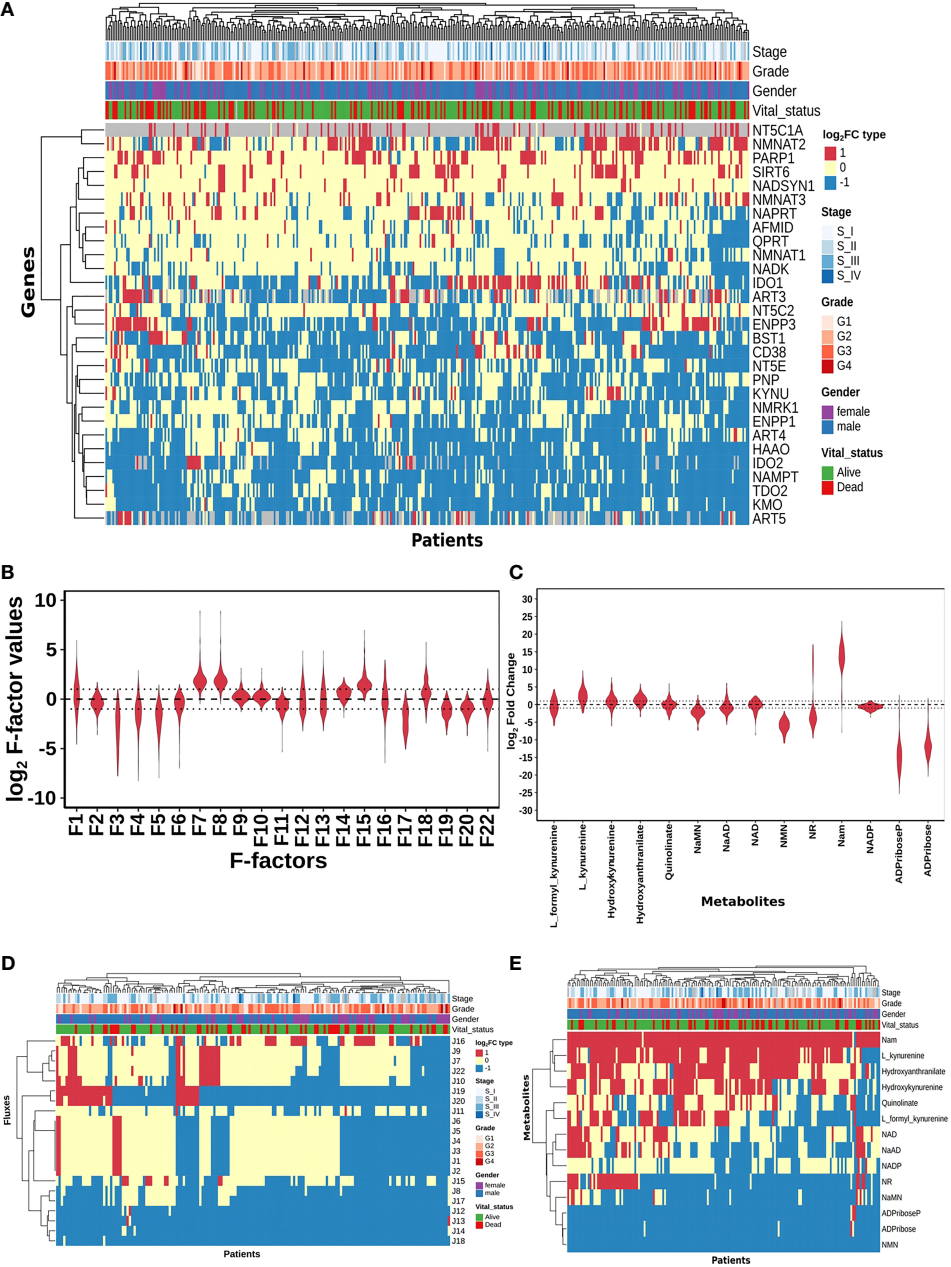
HCC patients exhibit heterogeneity in their NAD profiles. **(A)** Heatmap of NAD_net_ genes log_2_FC values in tumour tissue calculated with respect to the normal tissue, rows correspond to genes (n = 29) and columns correspond to patients (n = 371). The red colour represents the upregulation of gene expression in tumour tissue compared to the normal tissue (log_2_FC ≥ +1), the blue colour represents a downregulation of gene expression in tumour tissue compared to the normal tissue (log_2_FC ≤ -1), yellow colour represents no change of gene expression in tumour tissue compared to the normal tissue (-1 < log_2_FC < +1). Rows and columns are arranged based on the complete hierarchical clustering method. Annotations on the top of the heatmap are Stage, Grade, Gender, and Vital status (refer to the key in the image for more details). **(B)** Violin plot of log_2_ F-factor values of NAD_net_ model. The X-axis shows the F-factors and the Y-axis shows the log_2_ (fold change) values. **(C)** Violin plot of log_2_FC values of NAD_net_ metabolites. The X-axis shows the Genes, and the Y-axis shows the log_2_ (fold change) values. Metabolites are arranged according to the routes mentioned in **Figure 1A**. **(D)** Heatmap of reaction Fluxes obtained after steady-state analysis, rows correspond to reaction flux (n = 21), and columns correspond to patients (n = 168). The red colour represents an increase in flux compared to the base model, the blue colour represents a decrease in flux compared to the base model, and the yellow colour represents no change in flux compared to the base model. Rows and columns are arranged based on the complete hierarchical clustering method. Annotations on the top of the heatmap are Stage, Grade, Gender, and Vital status (refer to the key in the image for more details). **(E)** Heatmap of Metabolites obtained after steady-state analysis, rows correspond to metabolites and columns correspond to patients (n=168). The red colour represents an increase in the concentration of metabolite compared to the base model, the blue colour represents a decrease in the concentration of metabolite compared to the base model, yellow colour represents no change in concentration of metabolite compared to the base model. Rows and columns are arranged based on the complete hierarchical clustering method. Annotations on the top of the heatmap are Stage, Grade, Gender, and Vital status (refer to the key in the image for more details).

Our next goal was to build personalised NAD_net_ models for each HCC patient by integrating the transcriptomics data available in the TCGA-LIHC cohort. First, we studied the transcriptomic variation in the enzymes of the NAD_net_ in 365 patients in the dataset. The fold change of each gene for a patient sample was calculated by dividing the gene expression values of tumour tissue by the mean gene expression value of the normal liver tissues in the TCGA-LIHC cohort. Most of the NAD_net_ gene expressions in tumours were observed to be significantly different from the normal tissue (**Supplementary Tables 4, 5**). The distribution of log_2_FC gene expression values among the NAD_net_ genes was analysed, as shown in **Supplementary Figure 1A**, and heterogeneity among patients is shown in **Figure 2A**. To build personalised models, the fold change in expression value of each gene was converted as an expression factor (F1 - F22), which was further integrated into their corresponding reaction of the pathway (by utilising gene-protein-reaction association). As J1, J7, J8, J12, J13, J15, J16, and J18 reactions are associated with multiple genes, a cumulative sum of the fold change of genes associated with each reaction was considered as expression factors. For example, for the J1 reaction, the cumulative sum of *TDO2, IDO1*, and *IDO2* gene expression values was taken as the expression factor (F1).

A distribution of expression factors, shown in **Figure 2B**, clearly indicates high heterogeneity across the TCGA-LIHC cohort for this model. The variation was seen to be the highest for F7 and F8 (NMN adenylyltransferase), F15 (Mono ADPribosyltransferase), F1 (Tryptophan 2,3- dioxygenase), F18 (5’-Nucleotidase), F16 (NAD^+^ pyrophosphatase), F12 (NAD^+^ glycohydrolase), F13 (NAD(P)^+^ nucleosidase), and F22 (Nicotinate phosphoribosyltransferase) reactions (refer to **Supplementary Table 6**). The observed gene expression variations also suggest that there is likely to be variation in the reaction flux and the metabolite levels across different patients in the cohort (**Supplementary Figure S1A** and **Supplementary Table 7**). The mutation frequencies of the genes related to NAD_net_ were also obtained and analysed using the cBioPortal. The most frequent of them, which was in the *PARP1* gene, was seen to occur only in ~1% of the patients, while the rest of them were mutated in less than 1% of the patients (**Supplementary Figure S2**), clearly indicating that alterations in the NAD biosynthesis network are because of alteration in gene expression values, and not because of mutations.

#### Personalised NAD_net_ models indicate high patient heterogeneity in the dataset

The previous analysis (**Supplementary Figure 1**) reflected that the alterations in NAD_net_ profile in HCC could be attributed to variations in gene expression of the associated enzymes and also that there was no indication of any significant alteration in enzyme kinetics (K_m_, K_cat_). To construct personalised models for each HCC patient, the corresponding gene expression data was integrated into the base liver NAD_net_ as a surrogate measure of the enzyme abundance. Kinetic simulations and steady-state analysis of each personalised model were performed. Steady-state analysis was performed for all the models using different resolution thresholds. With the default COPASI resolution of 1E-09, we obtained 39 models with stable states. When the resolution threshold was lowered to 1E-03, we got 168 models obtaining stable states. With 1E-01 resolution threshold, we obtained 326 models. Considering the accuracy of defining a steady state and the number of models obtaining steady states, we used 1E-03 as the final resolution threshold. After steady-state analysis with the resolution threshold of 1E-03, 168 models out of 365 models were found to reach a stable state. As F-factors were the only difference among all the personalised models, the distribution of F-factors was compared between both the models, stable (models which obtained a stable state) and unstable (models which did not obtain a stable state) (refer to **Supplementary Figure S1B** and **Supplementary Table 7**). The unstable models had a significant difference in the values of F1, F2, F3, F12, F13, F14, F17, and F22 as compared to the stable models. Also, most of the F-factors distributions of the unstable models had a higher mean compared to the stable models. Notably, rate limiting reactions of the three routes of NAD synthesis, i.e., F1, F17, and F22, are significantly different and have higher values in the unstable models compared to stable models. Further, the NAD-consuming reactions F13 and F14 are also significantly higher in unstable models. Only the stable models are included for further analysis. Further, fold change values of each metabolite and reaction flux were calculated by dividing their respective steady-state values by the corresponding values in the NL model for the stable models. In the distribution of log_2_FC steady-state metabolites among stable models, shown in **Figure 2C**, all metabolites, except NADP, show high variance, clearly indicating high heterogeneity at the metabolite level as well (**Supplementary Table 8**).

A comparison of the steady-state concentrations of metabolites and the reaction fluxes in the pathway of individual HCC patients with that of NL revealed that the patients could be classified into three groups: (a) the pathway, on the whole, is downregulated, and the NAD pool is low (NAD_low), (b) the pathway, on the whole, is upregulated and the NAD level is high (NAD_high) and (c) the pathway does not show any significant change with respect to NL (NAD_No_change) (**Figures 2D, E**). The analysis clearly indicated that (a) the kynurenine pathway (Route I) was observed to be significantly downregulated or unchanged [J1, J2, J3, J4, J5, and J6] in all except six patients. (b) biosynthesis of NAD from NA (Route II) - [J22, J9, and J7] was found to be upregulated in one subset and downregulated in another subset of patients, while it is unchanged in all others (c) the salvage route of NAD biosynthesis (Route III) - [J18, J19, and J20] was also found to be upregulated in a subset of patients and downregulated in the rest (**Figure 2D**). These changes put together result in an accumulation of Nam in most patients. (d) Interestingly Route II and Route III are not upregulated together in any given patient (in one sub-subset of patients, Route II is upregulated, while in another Route III is upregulated), suggesting that upregulation of NAD biosynthesis occurs through different routes. Hierarchical clustering analysis of the fluxes and metabolites led to the identification of 4 clusters among the genes, largely corresponding to the route of NAD synthesis and utilisation, the fluxes, and metabolites among each route correlating positively within the same route (**Supplementary Figures S1C**, **S1D**).

### NAPRT levels are suggestive of NAD biosynthetic status

**Figure 3:**
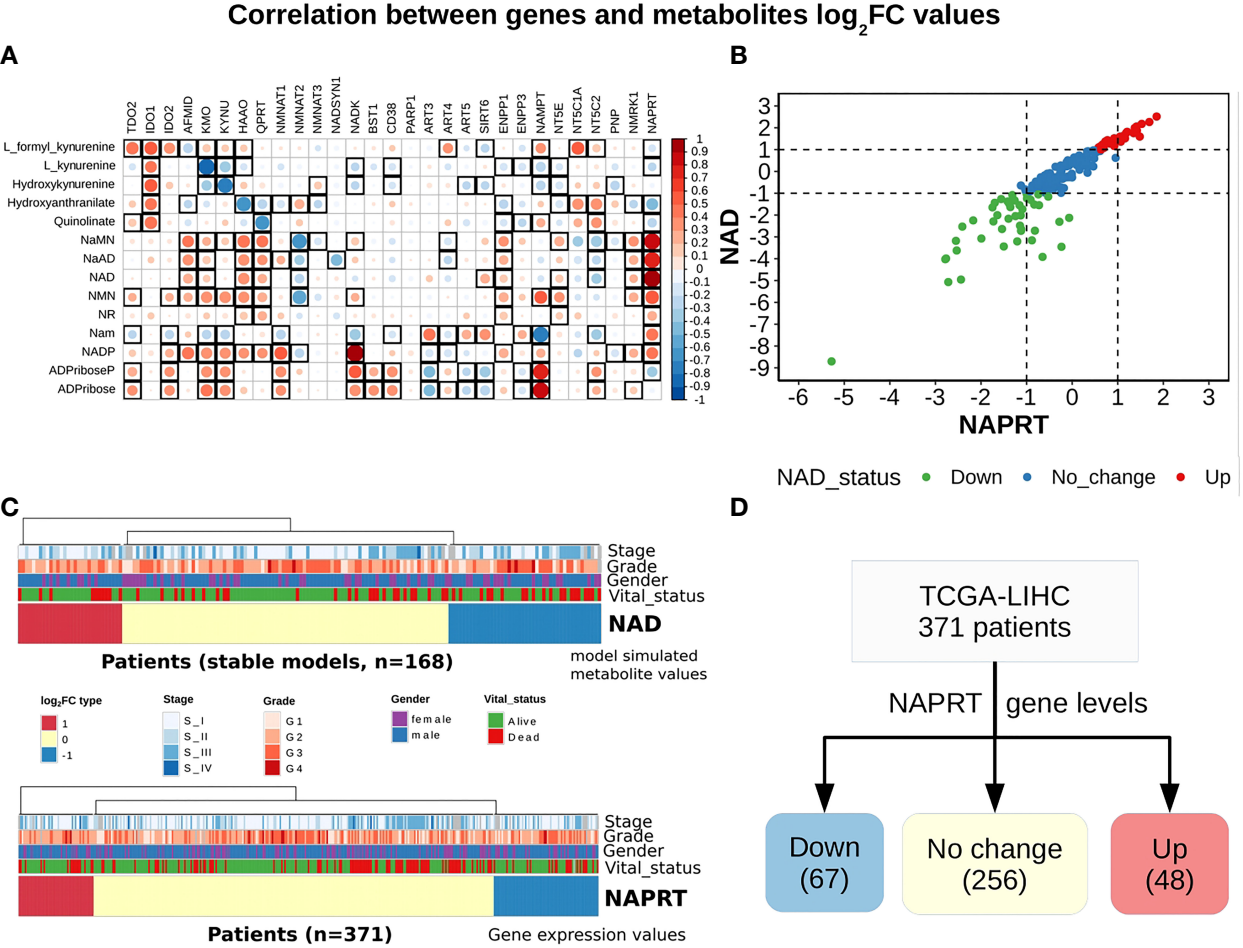
NAPRT alone is sufficient to indicate the NAD status in patients. **(A)** Correlogram between gene log_2_FC values and metabolite log_2_FC values. Rows represent metabolites and columns represent genes. The red colour corresponds to positive correlation, the blue colour corresponds to negative correlation, the area covered in the square corresponds to the absolute value of the correlation, and the black squares correspond to significant correlations (p-value < 0.05). Rows and columns are arranged based on the routes mentioned in **Figure 1A**. **(B)** Correlation plot showing the NAPRT and NAD log_2_FC values. The X-axis represents NAPRT log_2_FC values and the Y-axis represents NAD log_2_FC values. The points are coloured based on the NAD status of the samples. Red points indicate NAD up samples, blue points indicate NAD no change samples, and green points indicate NAD down samples. (n = 168) **(C)** Above panel shows the heatmap of NAD log_2_FC values obtained after steady-state analysis, rows correspond to NAD groups and columns correspond to patients (n = 168). The Red colour represents up NAD levels (log_2_FC ≥ +1), blue colour represents NAPRT down levels (log_2_FC ≤ -1), yellow colour represents NAD no change levels (-1 < log_2_FC < +1). The below panel shows the heatmap of NAPRT log_2_FC values in tumour tissue calculated with respect to the normal tissue, rows correspond to NAPRT groups and columns correspond to patients (n = 371). The Red colour represents up NAPRT levels (log_2_FC ≥ +1), the blue colour represents NAPRT down levels (log_2_FC ≤ -1), yellow colour represents NAPRT no change levels (-1 < log_2_FC < +1). Columns are arranged based on the complete hierarchical clustering method. Annotations on the top of the heatmap are Stage, Grade, Gender, and Vital status (refer to the key in the image for more details). **(D)** Schematic showing the division of patients into three groups based on NAPRT gene levels.

A steady-state concentration of a metabolite depends not only on the enzyme concentration but also on various other parameters such as the concentration of the input metabolite for the given reaction, K_m_, and K_cat_ of the enzymatic step, as well as on any feedback or feedforward loops for the given reaction. Therefore, metabolite abundance depends on the gene expression of the enzyme, metabolite inputs into the system, product metabolites, and the kinetics of the enzyme. The extent of correlation between the gene expression of all NAD_net_ genes and metabolites obtained after steady-state analysis was estimated for all patients in the TCGA-LIHC cohort using the Pearson correlation method (**Figure 3A**). Further, NAPRT was seen to have the highest correlation, which was statistically significant. The correlation values for all genes and metabolites, along with the statistical significance values [p-value and rho(r^2^)], are given in **Supplementary Table 9**. This study clearly demonstrated that NAD steady-state levels in a cell are correlated to the NAPRT gene expression (r^2 ^= 0.92) (**Figure 3B**), and therefore, NAPRT gene expression can be used as a readout for NAD biosynthesis in the cell. As NAPRT levels are indicative of NAD levels, all the patients can be grouped based on NAPRT levels into three groups a) NAPRT_Up group, where NAD levels are high as compared to normal liver, b) NAPRT_Down group, where NAD levels are low as compared to normal liver, and c) NAPRT_No_change group, where NAD levels are comparable with the normal liver (**Figures 3C, D**). The analysis also clearly shows that Route II is the critical determinant of NAD status in HCC patients.

### NAPRT is a control point in NAD_net_

**Figure 4:**
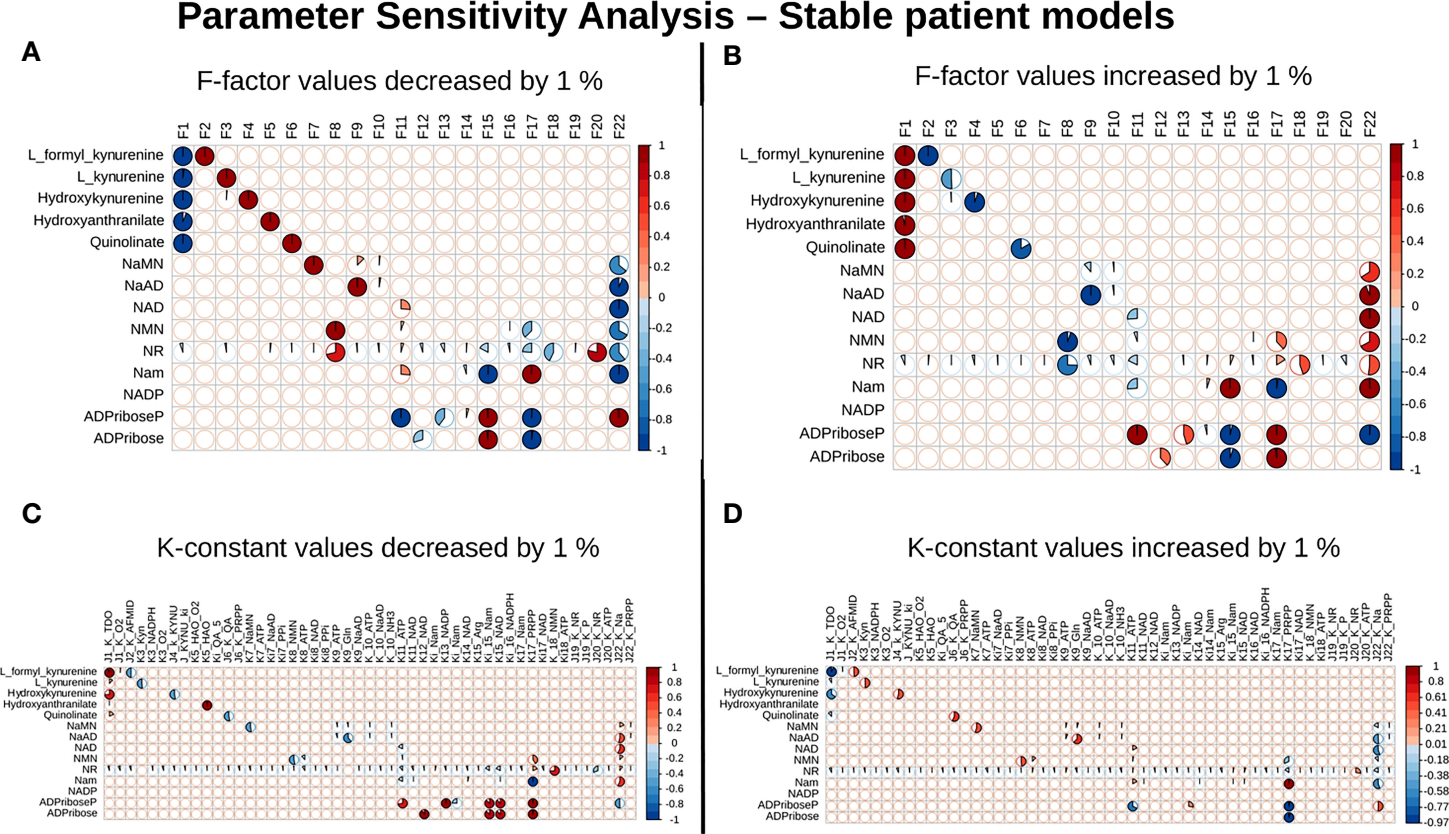
NAPRT is the control point in the NAD biosynthesis pathway in HCC patients. Correlogram between Metabolites and F-factors summarising the extent of patients affected with changes in the parameter values by -1 % in F-factors **(A)** by +1 % in F-factors **(B)** -1 % in K-constants **(C)**, and +1 % in K-constants **(D)**. The X-axis represents the parameters, and the Y-axis represents the metabolites. The red colour represents an increase in concentration and the blue colour represents a decrease in concentration. The area occupied by the coloured pie shows percentage of stable models, with greater than 1 % change in the concentration due to the change in the parameter value, out of 168 stable models.

Our next goal was to identify reactions that wielded the highest control on the NAD_net_, so as (a) to understand how the pathway dynamics are controlled and (b) to explore possible intervention points to manipulate the pathway. Further, it was of interest to investigate if the pathway control points varied significantly in different individuals in the cohort. Although the overall topology of the network remains the same, the weights associated with nodes (metabolites) and edges (reactions) change based on the gene expression patterns in different individuals, leading to the possibility of altering the control structures. To address this, the individual patient-wise kinetic models were used, and a parameter sensitivity analysis was performed on each of them using CoRC (refer to methods section parameter sensitivity analysis) and those reactions (and their corresponding genes) that had the highest influence on NAD levels were identified (**Figure 4** and **Supplementary Figure S3**). Each model parameter sensitivity was calculated as a percentage change from the unaltered model, and models showing greater than one percent are concerned as altered models. If any parameter had greater than +1 percent change, it was taken that it positively influences the metabolite concentrations, whereas parameters with less than -1 percent have a negative influence on the metabolite concentrations. F22 (NAPRT, (Nicotinate phosphoribosyltransferase)) was observed to have a positive influence on NaAD, NAD, and Nam metabolites, and F22 had a negative influence on ADPriboseP across all patients in the cohort. Therefore, an increase in the gene expression of the NAPRT gene will lead to enhanced levels of the NaAD, NAD, and Nam metabolites. F22 has a positive influence on NaMN, NMN, and NR in only a subset of patients. F11 (NAD^+^ Kinase) showed a negative influence on NAD in only a subset of patients but was not a control point in other patients. F1 (Tryptophan 2,3 dioxygenase) has a positive influence on the *de novo* pathway metabolites across all the patients in the cohort (**Figure 4** and **Supplementary Figure S3**). F1 is known to be the rate-limiting step of the *de novo* pathway (15), and the same was observed in our analysis. Further, even in the base model, F1, F17 and F22 were identified as the key factors controlling metabolite concentrations of their respective routes (**Supplementary Figure S3**).

### Survival analysis suggests potential benefits of NAD supplementation in NAPRT down subgroup

**Figure 5:**
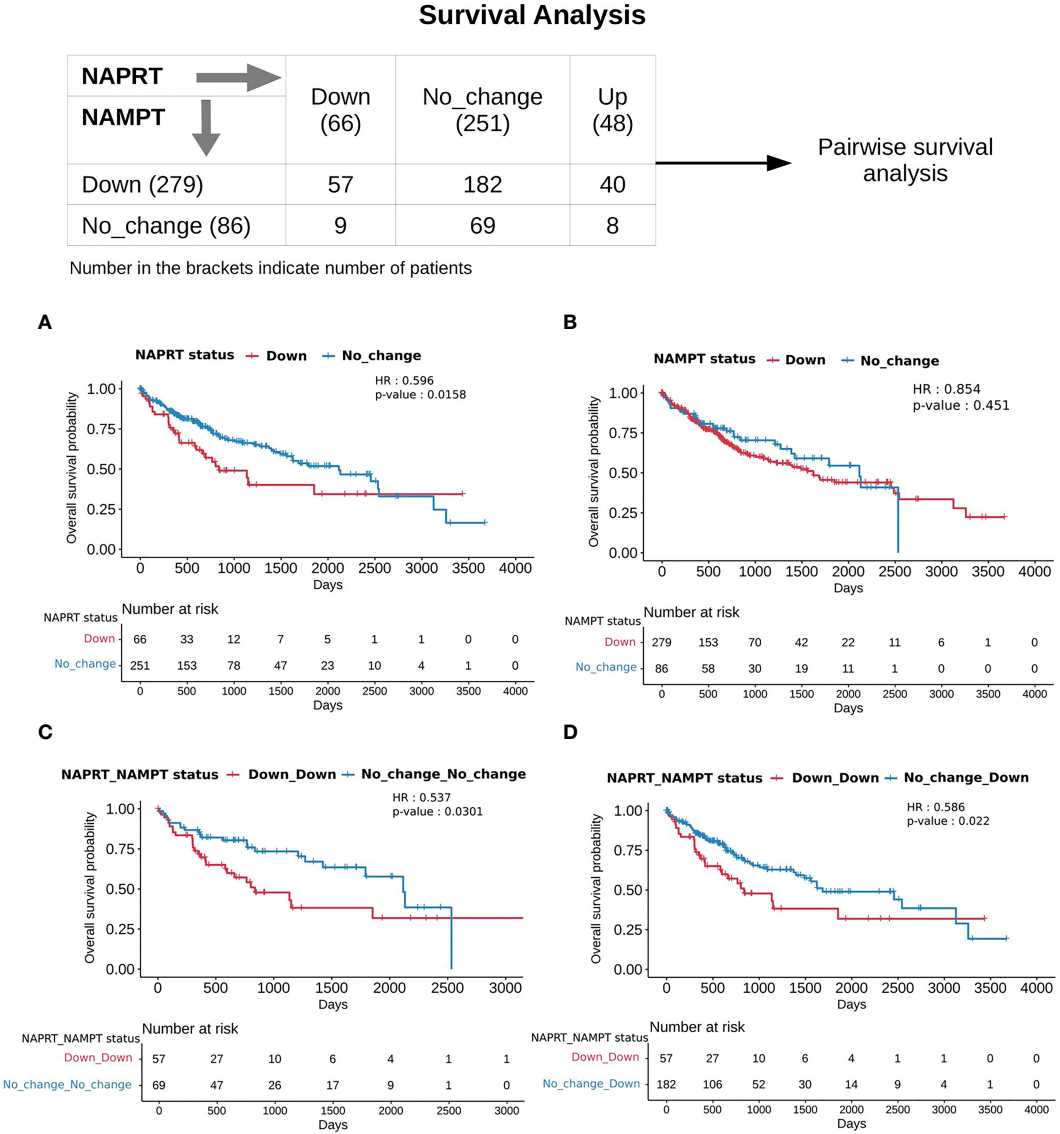
NAPRT_NAMPT Down_Down status corresponds to poorer survival. The panel on the top shows the distribution of patients into groups based on the NAPRT and NAMPT gene expression status. Kaplan-Meier Overall survival curve for HCC patients classified based on NAPRT Down and No_change groups **(A)** NAMPT Down and No_change groups **(B)**, NAPRT_NAMPT Down_Down versus No_change_No_change groups **(C)**, and NAPRT_NAMPT Down_Down versus Down_No_change groups **(D)**. HR and p-values reported in the figure panels are for the group represented in blue colour.

With the previous analysis, we identified NAPRT to be an indicator of NAD levels. We were interested in testing if there was any variation in survival in the groups based on NAPRT levels. For this, we performed a univariate cox-regression analysis using the predicted NAPRT level of individuals in the TCGA-LIHC cohort and calculated the extent of association of NAPRT level with HCC progression. Patients were divided into three groups based on NAPRT levels, NAPRT_Down (log_2_FC ≤ -1), NAPRT_No_change (-1 < log_2_FC < +1) and NAPRT_Up ( log_2_FC ≥ +1) (**Figure 3D**). A Kaplan-Meier analysis was performed, and a log-rank test was used to determine significant differences in the overall disease progression in all group pairs. We first tested if NAPRT levels by themselves have any prognostic value, but the correlation with the risk of patient mortality was non-significant when compared between NAPRT UP and Down subgroups (p-value = 0.407 and HR (Up) = 0.783) (**Supplementary Table 10**), but NAPRT Down subgroup was correlated significantly with patient mortality when compared with NAPRT No_change subgroup (p-value = 0.0158 and HR (No_change) = 0.596) (**Figure 5A**). NAMPT is known to be a poor prognosis marker and a known drug target for the NAD pathway in many cancers, and we tested if the levels of this gene had any prognostic value (49). Here too, we found the correlation with the risk of mortality to be non-significant (p-value = 0.451, HR (No_change) = 0.854) (**Figure 5B**). Clearly, neither NAPRT nor NAMPT did not have any survival prognosis by itself. We then tested if pairs of groups with different NAPRT and NAMPT statuses exhibited any survival difference. In total, six groups were tested (**Figure 5**). NAMPT did not have any upregulated patients in the TCGA-LIHC cohort; also, Route III was downregulated in most of the patients. Upon Kaplan-Meier analysis, we found that the NAPRT_NAMPT Down_Down group has a significantly poorer prognosis than other groups (**Figures 5C, D**; **Supplementary Table 10**). This suggests that, in the NAPRT_NAMPT Down_down group, the prognosis could be improved by NAD supplementation to improve survival.

## Discussion

Nicotinamide adenine dinucleotide (NAD), being an important cofactor in various biochemical reactions, plays a pivotal role in enabling and governing essential cellular activities. The levels of NAD are used by the cell as sensors for deciding what metabolic state it attains. A systems’ understanding of the pathways involved in NAD biosynthesis that provide quantitative insights is therefore important. The enzymes in the pathway have been well studied individually, and a wealth of biochemical information is available on each of them, enabling the reconstruction of a systems model of a NAD biosynthetic network (NAD_net_). We then use a kinetic modelling approach to study if there is variation in the NAD levels in HCC patients. Using NAD_net_ as a base model, we then construct personalised models for each patient by integrating with patient-specific gene expression values for all the enzymes in the network. While normal liver cells are known to use *de novo* NAD biosynthetic routes to maintain intracellular NAD levels, our model suggests that cancer cells are primarily dependent on the Preiss-Handler pathway (Route II in NAD_net_). NAPRT being a rate-limiting step of this route, is clearly seen to have altered gene expression in several HCC patients. While most studies provide population or cohort-level insights, our modelling approach of constructing personalised models has a unique advantage of providing insights at the individual patient level.

The effect of NAD on disease progression presents a complex picture. The analysis carried out here by studying perturbations at a patient level provides insights leading to sub-grouping. This, in turn, serves as a framework to resolve some of the inconsistencies evident in the literature. A subgroup of HCC patients with high NAD biosynthetic status responds differently to the subgroup that has low NAD biosynthetic status. While the first subgroup can be envisaged to benefit from an inhibitor of NAD biosynthesis, the latter subgroup will benefit from supplementation. Enhanced levels of NAD have been shown to support tumour proliferation. Inhibition of the pathway, specifically with NAMPT and NAPRT as drug targets, has been suggested as a strategy for reducing NAD levels (26). Both NAPRT and NAMPT are critical enzymes of two different routes of NAD synthesis. A combination of both gives a better representation of NAD levels. A group with NAMPT and NAPRT (Down_Down) group, has low levels of NAD, and they require supplementation; on the other hand, a group with NAMPT Down and other combinations of NAPRT (except NAPRT Down) can still maintain NAD levels. Our study shows that identifying the precise subgroup is essential for determining whether NAD inhibition or NAD supplementation would be beneficial.

Supplementation using readily available vitamin B3 supplements is an easy intervention to achieve if the subgroup is correctly identified. There are multiple lines of evidence in support of the supplementation. First, inhibition of NAD production has been associated with higher levels of DNA damage and triggering of hepatocarcinogenesis (28). Boosting NAD^+^ levels with supplements has been shown to have prophylactic effects in a genetically engineered mouse model of unconventional prefoldin RPB5 interactor (URI) used to study the mechanism of HCC development (28, 50). Second, in some other cancers, such as colorectal cancer, NAMPT and NAPRT high expression are seen to be associated with poor prognosis for the patient (51). Third, NAD levels were reported to be declining with age as well as implicated in a few liver diseases, including NAFLD (30). Due to this, several studies have proposed supplementation with NAD, and many NAD precursors are tested as supplements to increase NAD levels (52, 53). Among the precursors, Na was reported to be one of the best precursors with the least side effects and greater potential of getting converted into NAD (54).

The reconstructed model has the following three major limitations- (a) As the model has only biosynthesis reactions but not all the utilisation reactions of NAD, the model fails to capture the quantitative level of NAD in the cell, and (b) even though NAD metabolism is known to have subcellular compartmentalization of NAD pools both at the metabolite as well as the enzyme level; the reconstructed model considers the total NAD pool only, and there is no subcellular compartment in the model and therefore it cannot capture intracellular compartmental dynamics of NAD biosynthesis and (c) As metabolism is one of the most tightly regulated processes in the cell, regulatory interactions i.e., transcription factors that may govern the gene expression of enzymes of the NAD biosynthesis pathway are not included here, and therefore the effect of perturbation at transcription regulation cannot be modelled directly here.

Nevertheless, the model is useful for understanding the extent of variation in NAD biosynthesis at an individual patient level. From the correlation analysis, it is evident that the changes in gene expression are captured at the metabolite level. NAPRT levels are found to indicate the NAD biosynthetic status in the sample. Furthermore, NAPRT levels are regulated by MYC and TP53 transcription factors which are involved in cell growth and proliferation. NAPRT is also involved in immune and inflammation signalling (55).

In conclusion, we find high levels of heterogeneity in the NAD levels in HCC patients, and NAPRT gene expression levels are sufficient to indicate the NAD levels. Based on the NAPRT status, HCC patients can be subtyped into three categories corresponding to upregulation, no change, and downregulation of NAPRT with respect to a healthy liver. The NAPRT_Down group, when combined with NAMPT_Down, is seen to show poorer survival as compared to a group of HCC patients where the levels of these two enzymes are unaltered. Lower NAD levels correlate with lower levels of NAPRT and suggest that supplementation of NAD may be beneficial in this group of patients. Our study provides a rationale, and a means to explore subgrouping in HCC patients, paving the way for precision diagnosis and intervention.

## Data availability statement

The model generated in this study is deposited in EBI-BioModels and assigned the identifier MODEL2205250001: “https://www.ebi.ac.uk/biomodels/MODEL2205250001.1”;Scripts used in the study are made available on Github: “https://github.com/Adithya-C/NAD_LIHC_project”; Publicly available TCGA-LIHC dataset was analyzed in this study, which was retrieved from the Xena Browser, cohort: GDC TCGA Liver Cancer (LIHC): “https://xenabrowser.net/datapages/?cohort=GDC%20TCGA%20Liver%20Cancer%20(LIHC)&removeHub=https%3A%2F%2Fxena.treehouse.gi.ucsc.edu%3A443”.

## Author contributions

NC conceptualized and supervised the study. MM and OK constructed the initial computational model and AC refined the model. AC, MM, and SS performed the simulations. AC, MM, and NC analysed the results. AC, MM, and NC wrote the first draft of the manuscript. All authors have read and approved the final manuscript. All authors contributed to the article and approved the submitted version.

## Acknowledgments

We thank Department of Biotechnology (DBT), Government of India (Bioinformatics Centre Grant, BT/PR40187/BTIS/137/3/2021) for general support. AC acknowledges Council for Scientific Research (CSIR), Government of India for Senior Research Fellowship (SRF).

## Conflict of interest

NC is a co-founder of the companies qBiome Research Pvt. Ltd. and HealthSeq Precision Medicine Pvt. Ltd. They had no role in this manuscript.

The remaining authors declare that the research was conducted in the absence of any commercial or financial relationships that could be construed as a potential conflict of interest.

## Publisher’s note

All claims expressed in this article are solely those of the authors and do not necessarily represent those of their affiliated organizations, or those of the publisher, the editors and the reviewers. Any product that may be evaluated in this article, or claim that may be made by its manufacturer, is not guaranteed or endorsed by the publisher.

## Abbreviations

ADPR, ADP-ribose; cADPR, Cyclic ADP Ribose; COPASI,COmplex PAthway Simulator; CoRC, COPASI R Connector; DNA, Deoxyribonucleic acid; HCC, hepatocellular carcinoma; HR, Hazards ratio value; log_2_FC, log_2_ of Fold Change value; Na, Nicotinic acid; NaAD, Nicotinic acid adenine dinucleotide; NAD, Nicotinamide adenine dinucleotide; NAD_net_, quantitative kinetic model of the NAD biosynthetic pathway; NADP, Nicotinamide adenine dinucleotide phosphate; Nam, Nicotinamide; NaMN, Nicotinic acid adenine mononucleotide; NAMPT, Nicotinamide phosphoribosyl transferase; NAPRT, Nicotinate phosphoribosyl transferase; NAR, Nicotinic acid riboside; NL, normal liver; NMN, Nicotinamide mononucleotide; NMNAT, NMN adenylyltransferase; NR, Nicotinamide riboside; ODE, Ordinary differential equations; PRPP, Phosphoribosyl diphosphate; PARP, Poly (ADP-ribose) polymerase; PaxDb, Protein Abundance Database; RNASeq, RNA sequencing; ROS, reactive oxygen species; TCA, tricarboxylic acid cycle; TCGA-LIHC, The Cancer Genome Atlas-Liver Hepatocellular Carcinoma.
