# Supplementary material for: Personalized quantitative models of NAD metabolism in hepatocellular carcinoma identify a subgroup with poor prognosis
Source: Front Oncol. 2022 Sep 30;12:954512. doi: 10.3389/fonc.2022.954512 (PMC9565660; doi:10.3389/fonc.2022.954512)
Supplement: Supplementary file 2 [file DataSheet_2.pdf]

Supplementary Data

Supplementary Figures: 3

Supplementary Tables: 10

Supplementary Figure Legends

Figure S1

HCC patients exhibit heterogeneity in their NAD profiles at gene log_2FC levels and F-factor log_2FC levels

(A) Violin plot of log_2FC values of NAD_{net} genes. The X-axis shows the Genes and the Y-axis shows the log_2FC values. Metabolites are arranged according to the routes mentioned in **(Figure 1A).**

(B) Box plot of F-factor values of the TCGA-LIHC patients. The X-axis shows the F-factors, and the Y-axis shows the log_2 (F-factor) values. The green boxes represent models which obtained a stable state (n = 168) and the red boxes represent models which failed to obtain a stable state after the steady-state analysis (n = 198). The line in the middle of the box represents the median value, and the dots outside the boxes represent outliers. * Indicates t.test p-value < 0.05.

(C) Correlogram between reaction flux log_2FC values. Rows and columns represent reaction fluxes. The red colour corresponds to positive correlation, the blue colour corresponds to negative correlation, the area covered in the square corresponds to the absolute value of the correlation, and the black squares correspond to significant correlations (p-value < 0.05). The rows and columns are arranged according to the complete hierarchical clustering method

(D) Correlogram between metabolite log_2FC values. Rows and columns represent metabolites. The red colour corresponds to positive correlation, the blue colour corresponds to negative correlation, the area covered in the square corresponds to the absolute value of the correlation, and the black squares correspond to significant correlations (p-value < 0.05). The rows and columns are arranged according to the complete hierarchical clustering method.

Figure S2

Mutation analysis of TCGA-LIHC patient cohort for NAD_{net} genes

Heatmap of NAD_{net} genes and their mutation in TCGA-LIHC patient cohort. Rows represent genes and columns represent patients. The percentage of patients with mutation is displayed on the left of the heatmap. The rows are arranged based on the order of routes mentioned in NAD_{net} **(Figure 1A)**.

Figure S3

Base model parameter sensitivity analysis

Correlogram between Metabolites and F-factors summarizing the extent of change in the metabolite concentrations compared to the unperturbed model with changes in the parameter values by -1 % in F-factors **(A)**, by +1 % in F-factors **(B)**, -1 % in K-constants **(C)**, and +1 % in K-constants **(D)**. The X-axis represents the parameters, and the Y-axis represents the metabolites. The red colour squares represent an increase in concentration and the blue colour squares represent a decrease in concentration, and the black squares represent values with changes greater than 1 %. The area occupied in the square shows the percentage change in the concentration value compared to the unperturbed model.

Supplementary table captions

Supplementary Table 1: Full list of parameters used in the NAD_{net} model

Supplementary Table 2: List of fixed initial metabolite concentrations used in the model

Supplementary Table 3: Estimated F_{kcat} and V_{max}

Supplementary Table 4: Summary of t-test results between tumour and normal tissues for NAD_{net} genes, N_{normal} = 50, N_{tumour}= 371 patients of TCGA-LIHC cohort

Supplementary Table 5: Summary of log_2FC statistics for NAD_{net} gene for 371 patients of TCGA-LIHC cohort

Supplementary Table 6: Summary of log_2FC statistics for NAD_{net} F-factors for 371 patients of TCGA-LIHC cohort

Supplementary Table 7: Summary of t-test results between stable and unstable patient NAD_{net} models

Supplementary Table 8: Summary of log_2FC statistics for NAD_{net} metabolites for 168 stable patient models

Supplementary Table 9: Pearson correlation values and p-values between NAD_{net} genes and metabolites calculated from 168 stable patient models

Supplementary Table 10: Survival analysis between different groups of NAPRT and NAMPT patients
